# Memorial Service to the Late Miss Luckes

**Published:** 1919-03-01

**Authors:** 


### MEMORIAL SERVICE TO THE LATE MISS LUCKES.

### 22nd FEBRUARY, 1919.

A MEMORIAL SERVICE to the late Miss Luckes, Matron of the London Hospital, was held at the Hospital Church on Saturday last. The funeral itself had taken place at Golder's Green on the previous Wednesday. Few, however, of those who wished to do honour to Matron could attend the funeral; but every endeavour was made to let as many sisters and nurses attend the Memorial Service on Saturday as possible. The very beautiful Hospital Church of St. Philip seats about 500 people ordinarily, but from 900 to 1,000 were present at the Memorial Service. The members of the House Committee were present, and Lord Knutsford, the Chairman, represented Her Majesty Queen Alexandra, the President of the hospital, by her request.

The medical and surgical staff were present, and about 500 sisters and nurses of the hospital. The lay staff also attended. Most of the sister hospitals sent representatives; Dame Becher and Dame McCarthy, two old "Londoners," were there, representing the Army. Very many old personal friends of Miss Luckes attended, and we noticed Sir George Savage amongst these, and Lady Porter, wife of Sir James Porter, late Medical Director-General of the Admiralty.

The Service itself was beautiful and very simple. All the hymns were chosen because they were favourites of the late matron.

The service was conducted by the Lord Bishop of Stepney, Dr. Paget, the Rev. Sidney Vatcher, and the Rev. F. W. Heasman. The lesson was read by Lord Knutsford. Miss Muriel Foster, who never tires of giving her services to the "London," both in the wards for the benefit of the patients and at the services in the church, on this occasion added to the beauty of the service by singing "Oh, Rest in the Lord." The choir sang Tennyson's "Crossing the Bar" as an anthem.

The casket containing the ashes will be placed in a recess in the wall of the north aisle, over which a tablet will be fixed.

**Flowers were sent by Her Majesty, the Queen, to which was attached a card with the following inscription in the Queen's handwriting: "To the memory of dear Miss Luckes and in remembrance of her great and good work for the London Hospital.**

**Life's race well run,**
**Life's work well done,**
**Life's crown well won,**
**Now comes rest.**

**"ALEXANDRA."**

### The Memorial Service

TO

### EVA LUCKES.

### MATRON OF THE LONDON HOSPITAL, 1880 to 1919.

### Saturday, February 22, 1919, in the Hospital Church.

### ORDER OF SERVICE.

PROCESSIONAL.

Nearer, my God, to Thee,
Nearer to Thee;
E'en though it be a cross
That raiseth me;
Still all my song shall be,
Nearer, my God, to Thee,
Nearer to Thee;

(Hymns A. and M. No. 474.)

I am the Resurrection and the Life, saith the Lord; he that believeth in Me, though he were dead, yet shall he live: and whosoever liveth and believeth in Me shall never die.—St. John xi. 25, 26.

I know that my Redeemer liveth, and that He shall stand at the latter day upon the earth. And though after my skin worms destroy this body, yet in my flesh shall I see God: Whom I shall see for myself, and mine eyes shall behold, and not another.—Job xix. 25, 26, 27.

We brought nothing into this world, and it is certain we can carry nothing out. The Lord gave, and the Lord hath taken away; blessed be the Name of the Lord.—1 Tim. vi. 7. Job i. 21.

*Dixi, custodiam.* Psalm xxxix.

I said, I will take heed to my ways: that I offend not in my tongue.

I will keep my mouth as it were with a bridle: while the ungodly is in my sight.

I held my tongue, and spake nothing: I kept silence. yea, even from good words: but it was pain and grief to me.

My heart was hot within me, and while I was thus musing the fire kindled: and at the last I spake with my tongue.

Lord, let me know mine end, and the number of my days: that I may be certified how long I have to live.

Behold, thou hast made my days as it were a span long: and my age is even as nothing in respect to thee; and verily every man living is altogether vanity.

For man walketh in a vain shadow, and disquieteth himself in vain: he heapeth up riches, and cannot tell who shall gather them.

And now, Lord, what is my hope: truly my hope is even in thee.

Deliver me from all mine offences: and make me not a rebuke unto the foolish.

I became dumb, and opened not my mouth; for it was thy doing.

Take thy plague away from me: I am even consumed by means of thy heavy hand.

When thou with rebukes dost chasten man for sin, thou makest his beauty to consume away, like as it were a moth fretting a garment: every man therefore is but vanity.

Hear my prayer, O Lord, and with thine eyes consider my calling: hold not thy peace at my tears.

For I am a stranger with thee: and a sojourner, as all my fathers were.

O spare me a little, that I may recover my strength: before I go hence, and be no more seen.

Glory be to the Father, and to the Son: and to the Holy Ghost;

As it was in the beginning, is now, and ever shall be: world without end. Amen.

HERE FOLLOWED THE READING, BY THE LORD BISHOP OF STEPNEY, OF ONE OF HER FAVOURITE PASSAGES FROM THE NEW TESTAMENT.

"There cometh a woman of Samaria to draw water; Jesus saith unto her, Give me to drink."—1 St. John iv., 7.

My God, my Father, while I stray,
Far from my home, on life's rough way,
O teach me from my heart to say,
"Thy Will be done."

(Hymns A. and M. No. 425.)

### Viscount Knutsford, Chairman of the Hospital, Read the Lesson.

1 Cor. xv. 20.

Now is Christ risen from the dead, and become the first-fruits of them that slept. For since by man came death, by man came also the resurrection of the dead. For as in Adam all die, even so in Christ shall all be made alive. But every man in his own order: Christ the first-fruits; afterwards they that are Christ's, at his coming. Then cometh the end, when He shall have delivered up the kingdom to God, even the Father; when He shall have put down all rule, and all authority, and power. For He must reign, till He hath put all enemies under His feet. The last enemy that shall be

### THE LATE MISS LUCKES—(*continued*).

destroyed is death. For He hath put all things under His feet. But when He saith, all things are put under Him, it is manifest that He is excepted, which did put all things under Him. And when all things shall be subdued under Him, then shall the Son also Himself be subject unto Him that put all things under Him that God may be all in all. Else what shall they do which are baptized for the dead, if the dead rise not at all? Why are they then baptized for the dead? and why stand we in jeopardy every hour? I protest by your rejoicing, which I have in Christ Jesus our Lord, I die daily. If, after the manner of men I have fought with beasts at Ephesus, what advantageth it me, if the dead rise not? Let us eat and drink, for to-morrow we die. Be not deceived: evil communications corrupt good manners. Awake to righteousness and sin not; for some have not the knowledge of God. I speak this to your shame. But some man will say, How are the dead raised up? and with what body do they come? Thou fool, that which thou sowest is not quickened except it die. And that which thou sowest thou sowest not that body that shall be, but bare grain, it may chance of wheat, or of some other grain: but God giveth it a body, as it hath pleased Him, and to every seed his own body. All flesh is not the same flesh; but there is one kind of flesh of men, another flesh of beasts, another of fishes, and another of birds. There are also celestial bodies, and bodies terrestrial; but the glory of the celestial is one, and the glory of the terrestrial is another. There is one glory of the sun, and another glory of the moon, and another glory of the stars; for one star differeth from another star in glory. So also is the resurrection of the dead: It is sown in corruption; it is raised in incorruption: It is sown in dishonour; it is raised in glory: It is sown in weakness; it is raised in power: It is sown a natural body; it is raised a spiritual body. And so it is written, The first man Adam was made a living soul; the last Adam was made a quickening spirit. Howbeit that was not first which is spiritual, but that which is natural; and afterward that which is spiritual. The first man is of the earth, earthy: the second man is the Lord from heaven. As is the earthy, such are they that are earthy: and as is the heavenly, such are they also that are heavenly. And as we have borne the image of the earthy, we shall also bear the image of the heavenly. Now this I say, brethren, that flesh and blood cannot inherit the kingdom of God; neither doth corruption inherit incorruption. Behold, I shew you a mystery: We shall not all sleep, but we shall all be changed, in a moment, in the twinkling of an eye, at the last trump (for the trumpet shall sound), and the dead shall be raised incorruptible, and we shall be changed. For this corruptible must put on incorruption, and this mortal must put on immortality. So when this corruptible shall have put on incorruption and this mortal shall have put on immortality; then shall be brought to pass the saying that is written, Death is swallowed up in victory. O death, where is thy sting? O grave, where is thy victory? The sting of death is sin, and the strength of sin is the law But thanks be to God, which giveth us the victory through our Lord Jesus Christ. Therefore, my beloved brethren, be ye steadfast, unmoveable, always abounding in the work of the Lord, forasmuch as ye know that your labour is not in vain in the Lord.

Here the Clergy, accompanied by the Chairman of the Hospital, proceeded to the Shrine in the North Aisle, while the Choir sang the words of Lord Tennyson.

*Crossing the Bar.*

Sunset and evening star,
And one clear call for me!
And may there be no moaning of the Bar,
When I put out to sea,

But such a tide as moving seems asleep,
Too full for sound and foam,
When that which drew from out the boundless deep
Turns again home.

Twilight and evening bell,
And after that the dark!
And may there be no sadness of farewell,
When I embark;

For tho' from out our bourne of Time and Place
The flood may bear me far,
I hope to see my Pilot face to face
When I have crossed the Bar.

Man that is born of a woman hath but a short time to live, and is full of misery. He cometh up, and is cut down, like a flower; he fleeth as it were a shadow, and never continueth in one stay.

In the midst of life we are in death: of whom may we seek for succour, but of Thee, O Lord, Who for our sins art justly displeased?

Yet, O Lord God most holy, O Lord most mighty, O holy and most merciful Saviour, deliver us not into the bitter pains of eternal death.

Thou knowest, Lord, the secrets of our hearts; shut not Thy merciful ears to our prayer; but spare us, Lord most holy, O God most mighty, O holy and merciful Saviour, Thou most worthy Judge eternal, suffer us not, at our last hour, for any pains of death, to fall from Thee.

Forasmuch as it hath pleased Almighty God of his great mercy to take unto himself the soul of our dear Sister here departed, we therefore thankfully receive within the Walls of this Sanctuary of the great London Hospital the Casket containing Her Ashes as a most Sacred Trust and Treasure, to be by us and our successors for ever possessed and reverently guarded with loving care, so long as this consecrated edifice shall remain undestroyed; in sure and certain hope of the Resurrection to eternal life, through our Lord Jesus Christ; who shall change our vile body, that it may be like unto his glorious body, according to the mighty working, whereby he is able to subdue all things to himself.

"O Rest in the Lord"

Was sung by Miss Muriel Foster.

O Rest in the Lord, wait patiently for Him, and He shall give thee thy heart's desires. Commit thy way unto Him, and trust in Him, and fret not thyself because of evil doers. O Rest in the Lord, wait patiently for Him.

I heard a voice from heaven, saying unto me, Write, From henceforth blessed are the dead which die in the Lord; even so saith the Spirit; for they rest from their labours.

Lord, have mercy upon us.
*Christ, have mercy upon us.*
Lord, have mercy upon us.

Our Father, which art in heaven, Hallowed be thy Name. Thy kingdom come. Thy will be done in earth, As it is in heaven. Give us this day our daily bread. And forgive us our trespasses, As we forgive them that trespass against us. And lead us not into temptation; But deliver us from evil. Amen.

Almighty God, with Whom do live the spirits of them that depart hence in the Lord, and with Whom the souls of the faithful, after they are delivered from the burden of the flesh, are in joy and felicity; We give Thee hearty thanks, for that it hath pleased Thee to deliver this our *sister* out of the miseries of this sinful world; beseeching Thee, that it may please Thee, of Thy gracious goodness, shortly to accomplish the number of Thine elect, and to hasten Thy kingdom; that we, with all those that are departed in the true faith of Thy holy name, may have our perfect consummation and bliss, both in body and soul, in Thy eternal and everlasting glory; through Jesus Christ our Lord. *Amen.*

O Merciful God, the Father of our Lord Jesus Christ, Who is the Resurrection and the Life; in Whom whosoever believeth shall live, though he die; and whosoever liveth, and believeth in Him, shall not die eternally; who also hath taught us, by His Holy Apostle Saint Paul, not to be sorry, as men without hope, for them that sleep in Him; We meekly beseech Thee, O Father, to raise us from the death of sin unto the life of righteousness; that, when we shall depart this life, we may rest in Him, as our hope is this our sister doth; and that, at the general Resurrection in the last day, we may be found acceptable in Thy sight; and receive that blessing, which Thy well-beloved Son shall then pronounce to all that love and fear Thee, saying, Come, ye blessed children of my Father, receive the kingdom prepared for you from the beginning of the world; Grant this, we beseech Thee, O merciful Father, through Jesus Christ, our Mediator and Redeemer. *Amen.*

Lead, kindly Light, amid the encircling gloom,
Lead Thou me on;
The night is dark, and I am far from home,
Lead Thou me on.
Keep Thou my feet; I do not ask to see
The distant scene; one step enough for me.
(Hymns A. and M., No. 482)

The Benediction by the Lord Bishop of Stepney, followed by

Sung kneeling—

Peace, perfect peace, in this dark world of sin?
The Blood of Jesus whispers peace within.
(Hymns A. and M., No. 620).

Recessional—" Nunc Dimittis."

